# Letter to the Editor regarding ‘triglyceride glucose index: a significant prognostic marker of heart failure in patients with chronic kidney disease’

**DOI:** 10.1080/0886022X.2024.2448579

**Published:** 2025-01-06

**Authors:** Yao Yin, Si Jin

**Affiliations:** Department of Endocrinology, Institute of Geriatric Medicine, Liyuan Hospital, Tongji Medical College, Huazhong University of Science and Technology, Wuhan, China

We were intrigued by the article by Liu et al. [1] published in your journal *Renal Failure*, titled ‘Triglyceride Glucose Index: A Significant Prognostic Marker of Heart Failure in Patients with Chronic Kidney Disease’. The study found that elevated triglyceride-glucose (TyG) index levels were associated with an increased risk of cardiovascular death and heart failure readmission in patients with non-dialysis chronic kidney disease (CKD) stages 3–5, underscoring its prognostic value as an independent predictor of heart failure risk in this population. We commend the authors for their work and agree with their conclusions; however, we believe that several points warrant further discussion to enhance this important research.

Firstly, among the 284 patients included in the study, 109 had comorbid diabetes. Research indicates that in patients with type 2 diabetes mellitus (T2DM), the risk of major adverse cardiovascular events (MACE) increases with higher cumulative TyG index levels [2]. Furthermore, elevated TyG index levels in T2DM patients are associated with deterioration of left ventricular function and myocardial microvascular function [3]. This association may introduce bias into the study outcomes. Given the limited sample size for thoroughly investigating the relationship between the TyG index and CKD patients with diabetes, we recommend that the authors adopt stricter inclusion criteria or conduct subgroup analyses based on diabetes status.

Secondly, the TyG index, calculated as the product of fasting triglycerides and fasting glucose, is closely related to patients’ glucose and lipid profiles. Studies have shown that novel antidiabetic agents, such as liraglutide and dapagliflozin, can influence TyG levels [4]. Consequently, the varying medication regimens among the enrolled patients may introduce significant confounding factors. Therefore, it is crucial to perform stratified analyses based on the use of antidiabetic and lipid-lowering medications.

Finally, while the authors have commendably employed multiple methodologies, including electronic medical records and telephone follow-ups, to determine clinical endpoint events, certain potential factors remain underexplored. Lifestyle factors such as dietary changes and physical activity [5], along with socioeconomic status and psychological factors [6], can significantly impact cardiovascular disease (CVD) outcomes. Low levels of physical activity and unhealthy dietary habits are associated with an increased risk of cardiac metabolic risk factors. Additionally, socioeconomic status is a crucial determinant of health inequality; patients with low socioeconomic status (SES) face a higher risk of depression, and the combination of low SES and depression significantly elevates the risk of CVD and cardiovascular mortality. Future research should consider excluding individuals with extreme dietary habits, physical activity levels, or abnormal psychological characteristics, or incorporate these factors as covariates in stratified analyses. A deeper exploration of these variables will enhance our understanding of the observed disparities and bolster the reliability of the study findings.

In summary, we commend the authors for their insightful conclusions and valuable contributions. We look forward to future high-quality, large-scale studies that include diverse populations across various ages, ethnicities, and genders. Such research is essential to elucidate the functional relationship between the TyG index and the risk of CKD, as well as to determine the optimal TyG index thresholds for different patient subgroups.


Yao Yin and Si Jin *Department of Endocrinology, Institute of Geriatric Medicine, Liyuan Hospital, Tongji Medical College, Huazhong University of Science and Technology, Wuhan, China*
JinSi@hust.edu.cn


